# Narghile (water pipe) smoking among university students in Jordan: prevalence, pattern and beliefs

**DOI:** 10.1186/1477-7517-7-10

**Published:** 2010-05-24

**Authors:** Najla S Dar-Odeh, Faris G Bakri, Mahmoud K Al-Omiri, Hamzeh M Al-Mashni, Hazem A Eimar, Ameen S Khraisat, Shatha MK Abu-Hammad, Abdul-Aziz F Dudeen, Mohamed Nur Abdallah, Samer M Zied Alkilani, Louai Al-Shami, Osama A Abu-Hammad

**Affiliations:** 1 Dept. of Oral and Maxillofacial Surgery, Oral Medicine and Periodontology, Faculty of Dentistry, University of Jordan, Amman, Jordan; 2 Dept. of Internal Medicine, Faculty of Medicine, University of Jordan, Amman, Jordan; 3 Dept. of Prosthetic Dentistry, Faculty of Dentistry, University of Jordan, Amman, Jordan; 4 Dental Department, Jordan University Hospital, Amman, Jordan; 5 Dept. of Conservative Dentistry, Faculty of Dentistry, University of Jordan, Amman, Jordan

## Abstract

**Background and objectives:**

Narghile is becoming the favorite form of tobacco use by youth globally. This problem has received more attention in recent years. The aim of this study was to investigate the prevalence and pattern of narghile use among students in three public Jordanian universities; to assess their beliefs about narghile's adverse health consequences; and to evaluate their awareness of oral health and oral hygiene.

**Methods:**

The study was a cross-sectional survey of university students. A self-administered, anonymous questionnaire was distributed randomly to university students in three public Jordanian universities during December, 2008. The questionnaire was designed to ask specific questions that are related to smoking in general, and to narghile smoking in specific. There were also questions about oral health awareness and oral hygiene practices.

**Results:**

36.8% of the surveyed sample indicated they were smokers comprising 61.9% of the male students and 10.7% of the female students in the study sample. Cigarettes and narghile were the preferred smoking methods among male students (42%). On the other hand, female students preferred narghile only (53%). Parental smoking status but not their educational level was associated with the students smoking status. Smokers had also significantly poor dental attendance and poor oral hygiene habits.

**Conclusion:**

This study confirmed the spreading narghile epidemic among young people in Jordan like the neighboring countries of the Eastern Mediterranean region. Alarming signs were the poor oral health awareness among students particularly smokers.

## Introduction

Jordan is a small country located in the Eastern Mediterranean region (EMR). It has a relatively small population of about 5,600,000. Almost half of this population is comprised of adolescents and youth. Like many countries of the EMR, Jordan is affected by the tobacco epidemic. A recent survey by the Jordanian Ministry of Health found that smoking increased from 27-29% among Jordan's population over the period from 2005 to 2007[1]. Moreover, a 2006 study found that 13.6% of youth, aged 13-15, smoked cigarettes, and 22.7% opted for the narghile[1]. According to Warren et al (2009) current cigarette smoking decreased significantly for boys and girls in Jordan (2003 to 2007) due to implementation of tobacco control policies, on the other hand, other tobacco use increased significantly for boys and girls in Jordan (1999 to 2007)[2].

One of tobacco smoking methods that is appealing to the young is the narghile. The use of narghile by the young was attributed to the positive sensory characteristics of narghile like the attractive smell and taste [3]. Younger members of the community including women are being encouraged to use this method of smoking under the misconception of its safety compared with smoking cigarettes [4].

It is believed that there is a narghile's current surge in popularity in the EMR [3] despite the many efforts for controlling the tobacco epidemic. Narghile smoking has been linked to a variety of adverse health effects. Aldehyde compounds found in narghile smoke are known to be toxic, carcinogenic and hazardous [5]. By contrast to cigarette smoking, one session of narghile smoking is thought to release greater amounts of formaldehyde, acetaldehyde, acrolein propionaldehyde and methacrolein [5]. Systemically, narghile smoking is associated with decreased pulmonary function leading to increased risk of chronic obstructive airway disease [6,7]. Furthermore, there have been reports of an association with certain types of cancer including bronchogenic carcinoma[8], oesophageal carcinoma[9], bladder cancer[10], and pancreatic cancer[11].

Significantly few studies have reported the effects of narghile smoking in the oral cavity[12]. The habit is known to promote periodontal bone loss[13], and increase the possibility of acute osteitis (dry socket) after tooth extraction [14,15]. The positive correlation between tobacco smoking in general and increased oral carriage of *Candida *species has also been reported [16-19].

Assessing practices and opinions related to narghile among university students represents an important starting point, to increase understanding of future trends, and possible ways to curb the spread of narghile use [3]. Furthermore, it is important to understand its use patterns and dependence-producing characteristics to develop successful, culturally appropriate prevention and cessation strategies[20].

A number of surveys have been conducted to investigate tobacco use among Jordanian university students [21,22]. However, none of them focused on the beliefs of students regarding narghile use or investigated their awareness regarding oral health or oral hygiene. On the other hand, many surveys investigated narghile use among university students in the neighboring countries [3,20,23,24], and more recently in the western countries [25-28]. These studies provide an evidence of increased popularity of narghile among this young sector of the population.

The aims of this study are: to investigate the prevalence and pattern of narghile use among students in three public Jordanian universities; to assess their beliefs about narghile's adverse health consequences; and to evaluate their awareness of oral health and oral hygiene.

## Methods

### Data collection

The study was a cross-sectional survey of university students. A self-administered, anonymous questionnaire was distributed randomly to university students in three public Jordanian universities during December, 2008. Three teams (each comprised of two) were distributed to the investigated universities. It was decided to investigate universities located in the north, middle and south of the country. Hence, the universities investigated were (from north to south), Jordan University of Science and Technology (JUST) in Irbid, University of Jordan (UJ) in Amman, and Mu'tah University (MU) in Karak. Questionnaires were distributed randomly to students on campus, and the distributing teams were available during filling-up the questionnaire, which was written in Arabic to explain questions in case they were unclear. The questionnaire was designed to ask specific questions that are related to smoking in general, and to narghile smoking in specific. There were also questions about oral health awareness and oral hygiene practices.

### Statistical analysis

The Statistical analysis program SPSS (Statistical Package for Social Sciences) was used to indicate the significant differences between groups.

## Results

### Characteristics of smokers

The number of students at the time of survey in the three universities as provided by the relevant departments of admission and registration were as follows: 38,690 at the UJ, 20,409 at JUST and 17,000 at MU.

A total number of 1454 students were included in the study with 741 males and 712 females (one student failed to state the gender). 3 students failed to indicate their university; hence, there were 495 students from UJ (1.3% of UJ students), 479 students from JUST (2.35% of JUST students) and 477 students from MU (2.8% of MU students).

Age range was 16-26 years. A total of 535 students (36.8% of the surveyed sample in the three universities) indicated they were smokers with 459 males (61.9% of the total male students) and 76 females (10.7% of the total female students). When cross-tabulated, these data indicated highly significant difference in the incidence of smoking between males and females (P = 0.0001).

The total number of students who declared they were non-smokers was 919. However, eight of them reported they smoke cigarettes daily, 37 reported they smoked cigarettes on non-daily basis, 37 also reported they smoke narghile only, and 3 reported they smoke narghile and cigarettes.

Cigarettes and narghile were the preferred smoking methods among male students (42%). On the other hand, female students preferred narghile only (53%). (Table 1)

**Table 1:** Forms of tobacco used by smoker students (admitters and deniers) according to gender.

Gender	Cigarettes only No (%)	Narghile only No (%)	Both cigarettes & narghile No (%)	Total
Males	181(37%)	106(21%)	210 (42%)	497
Females	39 (33%)	63(53%)	17(14%)	119

The large number of female students smoking narghile only was found to be significantly higher than those smoking cigarettes alone or in association with narghile when these data were cross tabulated with the numbers of male and female smokers (p < 0.0001).

**Figure 1:**
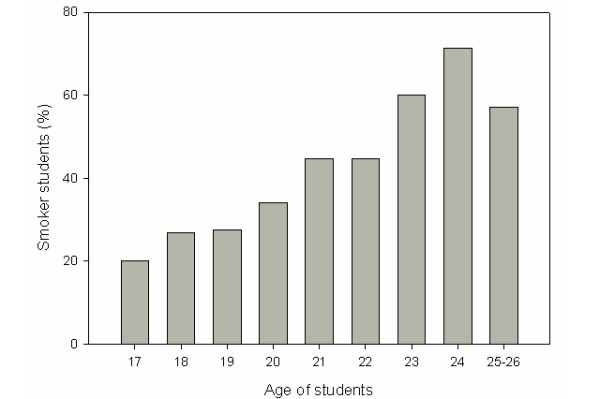
**Percentages of smoker students with regards to age**.

Regarding the age of smokers, it was found that smokers constituted 20% (n = 1) of the 17 year-olds, 26.8% (n = 64) of the 18-year olds, and 27.5% (n = 86) of the 19-year olds, 34.1% (n = 118) of the 20-year olds, 44.8% (n = 116) of the 21-year olds, 44.8%(n = 78) of the 22-year olds, 60% (n = 45) of the 23-year olds, 71.4% (n = 15) of the 24-year olds, and 57.1% (n = 12) of the 25-26-year olds (Figure 1).

There were 1373 individuals who gave information both on: their own and their parents' current smoker status. Within this group of students there were: 501 non smoking students with non smoking parents, 357 non smoking students who had smoking parents, 261 smoking students who had smoking parents while 254 smoking students who had non-smoking parents. These data when cross-tabulated show significant association between the students' smoking status and their parents' smoking status with P = 0.0012.

Regarding the parents' level of education, It was found that: 49 smokers and 70 non-smokers had illiterate parents, 142 smokers and 250 non-smokers had parents with school (unspecified level) education, 253 smokers and 468 non-smokers had parents with university level of education and 66 smokers and 107 non-smokers had parents with education level higher than the bachelors degree. 25 smokers and 24 non-smokers withheld information about their parents' level of education. When these data were cross tabulated, the chi-square test indicated insignificant level of association between the smoking status and the parents' level of education with P = 0.59.

When the effect of place of residence on the number of smoker students was tested by cross tabulating the numbers of residents of rural/urban areas, with the numbers of smokers/non-smokers, it was found that 340 smokers resided in urban areas, 179 smokers resided in rural areas, 629 non-smokers resided in urban areas and 268 non-smokers resided in rural areas (16 smoking and 22 non-smoking withheld information about residence). The results indicated non-significant association of the place of residence and the students' smoking status with P = 0.74.

### Characteristics of narghile smokers

Although only 396 students stated they were current narghile smokers, a total of 552 students indicated how frequent they use narghile; 72 students indicated they smoked narghile on a daily basis, 297 students indicated they smoked narghile on a weekly basis while 183 students indicated they smoked narghile only occasionally.

Only 512 students indicated the age of onset of narghile smoking. Two students indicated they smoked at 8, one student at 9, 15 students at 10, 2 students at 11, 15 students at 12, 8 students at 13, 24 students at 14, 41 students at 15, 91 students at 16, 81 students at 17, 114 students at 18, 57 students at 19, 42 students at 20, 12 students at 21, 5 students at 22 and 2 students at 23

158 students indicated they usually have narghile at home, 359 students indicated they usually have it at coffee shops and 158 students indicated they usually have it somewhere else.

438 students indicated they started smoking narghile with friends, 67 with family and 3 with friends and family. At present, 493 students indicated they smoke narghile with friends, 67 with family while no body reported they are currently smoking narghile with family and friends.

237 students indicated they do not use their own hose tips when smoking narghile, while 288 students indicated they use their own hose tips.

### Beliefs about adverse health effects of smoking and oral hygiene practices

Only 143 students thought smoking narghile is more harmful than cigarette smoking while 1165 thought it was not.

The most frequently stated harmful effects of narghile were: Respiratory diseases (540), cancer (503), cardiovascular disease (291), and mouth disease (85).

Only 42 out of the 535 (8%) of smoker students claimed that they were regular attendees for their dentists, while the majority stated that they visit the dentist only when they have pain, on irregular basis or gave no comment on the question.

On the other hand 110 (12%) non smoker students were regular visitors to the dentist, while 809 (88%) visited their dentists when they had pain or admitted being irregular attendants, or gave no comments. Cross tabulation of these data revealed a significant association between smoking status and attitude towards visiting the dentists with a p value of 0.04.

Oral hygiene habits of smokers and non smokers are shown in Table 2.

**Table 2:** Oral hygiene habits of smokers and non-smokers.

Oral hygiene habits	Smokers [no (%)]	Non smokers [no (%)]
Don't brush	42(8%)	29 (3%)

Brush once daily	294 (55%)	278 (30%)

Brush twice or more	199 (37%)	612 (67%)

Use dental floss	76 (14%)	152 (17%)

## Discussion

The sample of university students sharing in this study were randomly selected from three public universities covering all parts of the country. This was to ensure that the sample was as representative as possible. None of the private universities was included; however, socioeconomic background of students was not expected to be different between both types of universities, since public universities have a system similar to private ones in acceptance of students. It was also aimed to involve students from almost all faculties including medical ones, since those are supposed to have a better knowledge regarding health hazards of tobacco use.

The relatively high percentage of current narghile smokers (25% use narghile on a daily or weekly basis) is comparable to that of neighboring countries[3,24]. A much less percentage was reported in Western Europe[27]. Another trend that was also noted in neighboring countries is that: age and sex prevalence of tobacco smoking in general favored older students and male students[20].

The majority of female smokers, smoke narghile, either exclusively (53%), or to a lesser extent, in association with cigarettes (14%). The female preference of narghile reflects the social taboo against cigarette smoking by women in conservative societies in the region[29]. On the other hand, the majority of male students preferred cigarettes either exclusively (37%) or in association with narghile (42%). Narghile smoking is becoming more prevalent among women and girls in the Eastern Mediterranean region because of more lax family and social attitudes related to it [23]. Recent studies from Lebanon show less gender difference in the prevalence of cigarette smoking and even higher prevalence rates of narghile smoking compared to cigarettes among females [24,30].

Social attitudes are also reflected on the initiating age of smoking. Some of students in this sample started smoking narghile during their adolescence and even childhood. Although most of the ever narghile smokers started the habit in company with friends, some of them actually started the habit with a family member, which stresses the role of family in formulating unwanted social habits like smoking. However, peer influence appears to be an equally important, or even a more important determinant of the smoking habits of university students [3], where a friend was most often the introducer, motivator, and companion for smoking [31]. This can explain why most of our narghile users, practice this habit at coffee shops rather than their homes. The spread of a large number of coffee shops serving narghile in Jordan has definitely provided a characteristic social atmosphere for youth of both genders to enjoy without much (if any) parental opposition. Narghile smoking is embedded in Arab culture, and sharing narghile provides a means of demonstrating the hospitality and generosity characteristic of an adult Arab male[32].

An alarming sign was that a substantial proportion of narghile smokers (45%) not only share the narghile but also the hose tip which could be a substantial source of cross infection.

Whereas smoking status of students was strongly associated with smoking parents, their parents' level of education was not. This was in contrast to data from university students in Lebanon which shows that smoking is related to parental education, suggesting the influence of socioeconomic status on the smoking behavior of youth [30].

Again, place of residence whether rural or urban had no influence on the smoking status; this is probably because the borders between both areas are starting to disappear under the influence of globalization and modernization.

Based on the findings of this study, some students (9% of the non-smoking students) might perceive themselves as non-smokers; still they actually use tobacco in one form or another. Denial behavior in smoking was reported previously among university students who might consider themselves as social smokers but not smokers [33].

Interestingly, a substantial proportion of students (89%) thought that narghile is less harmful than cigarettes, in contrast to their peers in a neighboring country [3]. However, students in both countries provided the same adverse effects of narghile; namely, respiratory disease, cancer, and cardiovascular disease [3].

In this study, the fourth adverse effect of narghile in terms of frequency was mouth disease; namely, dental caries, gingivitis and halitosis. This reflects the increased awareness of students regarding oral disease and oral hygiene.

Nevertheless, a significantly higher proportion of non smokers attend their dentist's compared to smokers.

The higher awareness of non smokers of oral health is also manifested by their better oral hygiene habits as shown by the higher percentage of those who brush twice or more daily and who floss their teeth.

Unfortunately, the neglected oral hygiene and the irregular dental attendance shown clearly by students particularly the smoker ones may reflect adversely on oral health. It is well-established that the dental office provides an excellent venue for providing tobacco intervention services[34], and it also helps in the primary prevention of oral cancer which all smokers certainly need.

Despite the controversy associated with potential health hazards of narghile, one cannot overlook the many ways narghile can be even more harmful than cigarettes. There is the hazardous social aspect of narghile smoking that involves sharing of family members including the wife and children, or friends getting together in a coffee shop for instance. In Jordan the law of public health for the year 1977 prohibits smoking in public places [35], however, no action is being done to counteract the continuous establishment of new coffee shops serving narghile to youth. Whether associated health hazards are overestimated by our respondents or not, the dark side becomes more obvious with the increasing involvement of vulnerable part of the population namely young people and women.

Research in the field of adverse effects of narghile on the oral tissues is mostly outdated [36,37]. More recent research is either directed towards less serious effects of narghile [13,14], or towards the effects of narghile in association with other forms of tobacco use and not narghile exclusively [12-14]. More research is needed to investigate adverse health effects of narghile on the oral tissues particularly the potential carcinogenic effect.

This becomes even more crucial when fighting an epidemic that seems to affect people of all ages and nations.

## Competing interests

The authors declare that they have no competing interests.

## Authors' contributions

ND Participated in the design of the study, coordination and helped to draft the manuscript; FB Participated in the study design; MA Helped in statistical analysis and reviewed the manuscript; HA Prepared the review of literature on the subject; HE Prepared the review of literature on the subject; AK Reviewed the manuscript; SA Prepared the review of literature on the subject; AD Participated in statistical analysis; MA Participated in statistical analysis; SZ Participated in statistical analysis; LA Participated in statistical analysis; OA Participated in drafting the manuscript, statistical analysis. All authors, read and approved the final manuscript.
